# A Social Analgesic? Acetaminophen (Paracetamol) Reduces Positive Empathy

**DOI:** 10.3389/fpsyg.2019.00538

**Published:** 2019-03-29

**Authors:** Dominik Mischkowski, Jennifer Crocker, Baldwin M. Way

**Affiliations:** ^1^ Department of Psychology, Ohio University, Athens, OH, United States; ^2^ Ohio Musculoskeletal and Neurological Institute, Ohio University, Athens, OH, United States; ^3^ Department of Psychology, The Ohio State University, Columbus, OH, United States; ^4^ Institute for Behavioral Medicine Research, The Ohio State University, Columbus, OH, United States

**Keywords:** acetaminophen, paracetamol, positive empathy, cyberball, psychopharmacology

## Abstract

Acetaminophen – a potent physical painkiller that also reduces empathy for other people’s suffering – blunts physical and social pain by reducing activation in brain areas (i.e. anterior insula and anterior cingulate) thought to be related to emotional awareness and motivation. Some neuroimaging research on positive empathy (i.e., the perception and sharing of positive affect in other people) suggests that the experience of positive empathy also recruits these paralimbic cortical brain areas. We thus hypothesized that acetaminophen may also impair affective processes related to the experience of positive empathy. We tested this hypothesis in a double-blind, placebo-controlled experiment. Specifically, we administered 1,000 mg acetaminophen or a placebo and measured effects on different measures of positive empathy while participants read scenarios about the uplifting experiences of other people. Results showed that acetaminophen reduced personal pleasure and other-directed empathic feelings in response to these scenarios. In contrast, effects on perceived positivity of the described experiences or perceived pleasure in scenario protagonists were not significant. These findings suggest that (1) acetaminophen reduces affective reactivity to other people’s positive experiences and (2) the experience of physical pain and positive empathy may have a more similar neurochemical basis than previously assumed. Because the experience of positive empathy is related to prosocial behavior, our findings also raise questions about the societal impact of excessive acetaminophen consumption.

Experiencing and expressing positive empathy for other people’s good fortunes have striking personal and interpersonal benefits (Morelli et al., 2015). A substantial amount of research shows that understanding and sharing in other people’s pleasurable experiences foster psychological health, interpersonal trust, intimacy, and a prosocial orientation, both for the source and the recipient of positive empathy (Smith et al., 1989; Gable et al., 2006; Sallquist et al., 2009; Reis et al., 2010; Morelli et al., 2015; Andreychik and Lewis, 2017). Despite this host of findings on the psychological and relational benefits of positive empathy, however, our understanding of the physiological bases of positive empathy remains limited.

Most recent research on the biological underpinnings of positive empathy has focused on pinpointing its brain correlates using functional magnetic resonance imaging (fMRI; e.g., Immordino-Yang et al., 2009; Mobbs et al., 2009; Morelli et al., 2014; Lamm et al., 2015; Lockwood, 2016; Chiesa et al., 2017). Across fMRI studies, the experience of positive empathy seems to be represented in a complex neuronal network involved in emotional awareness, shared affect, and vicarious reward (e.g., Immordino-Yang et al., 2009; Ebisch et al., 2011; Braams et al., 2013; Apps and Ramnani, 2014; Lamm et al., 2015; Morelli et al., 2015). These studies have shown that positive empathy has a distinct neuronal signature compared to related psychosocial affective experiences, such as personal reward or negative empathy, which is generally defined as responsiveness to other people’s pain (Immordino-Yang et al., 2009; Ebisch et al., 2011; Morelli et al., 2014, 2015; Lamm et al., 2015; Lockwood, 2016). fMRI can contribute to our understanding of the component psychological processes of positive empathy but constitutes a correlational methodology, which limits our ability to make causal conclusions about the different processes underlying empathy (Decety, 2010; Zaki et al., 2016; Lamm et al., 2017).

Pharmacological experiments can supplement existing correlational findings about the nature of empathy, because such studies add a causal approach to the study of empathy’s psychological underpinnings (e.g., Rütgen et al., 2015, 2017; Mischkowski et al., 2016). Recent research suggests that the painkiller acetaminophen (or paracetamol, under its international designation) may be particularly well suited for studying the psychology of empathy, as acetaminophen reduces empathy for pain (Mischkowski et al., 2016). Acetaminophen is one of the most popular medicines in the USA (Kaufman et al., 2002) and easily accessible over the counter. Furthermore, acetaminophen is a potent analgesic, reducing pain in response to heat, electric shock, or cold (e.g., Bromm et al., 1992; Nielsen et al., 1992; Piguet et al., 1998; Yuan et al., 1998; Pickering et al., 2015). In addition to these effects on pain, acetaminophen also reduces psychological reactivity (e.g., DeWall et al., 2010; Randles et al., 2013, 2016; Durso et al., 2015; Roberts et al., 2017). Thus, acetaminophen affects a broad spectrum of psychological processes that are not limited to the processing of physical pain.

Neuroimaging evidence suggests that key brain areas involved in these psychological effects of acetaminophen are likely to be the anterior insula (AI) and anterior parts of the cingulate cortex (dACC). Acetaminophen reduces activation in these areas during physical and emotional pain (DeWall et al., 2010; Pickering et al., 2015). Some researchers have pointed out the centrality of these brain areas for positive empathy, as well (e.g., Immordino-Yang et al., 2009; Apps and Ramnani, 2014; Lockwood, 2016). This shared neural mechanism is plausible as the AI seems to be the core of a limbic cortical network related to emotional awareness, independent of emotional valence (Craig, 2009). Thus, both positive and negative empathy may rely on AI and ACC, even though these types of empathy are differentiable at other levels along the neuroaxis. Because acetaminophen appears to blunt responsiveness for one’s own pain and for the pain of others in brain areas overlapping with those involved in positive empathy, we hypothesize that acetaminophen may also impair people’s ability to experience empathy for others’ positive experiences.

To test this hypothesis, we used unpublished data from a previously published dataset (Study 2; Mischkowski et al., 2016). We administered acetaminophen or an inert placebo to healthy volunteers and tested participants’ empathic responses when reading written scenarios about different protagonists having uplifting, positive experiences. To further probe the effect of acetaminophen on positive empathy, we distinguished between empathic perceptions and empathic affect when measuring positive empathy (e.g., Davis, 1994; Decety and Jackson, 2004; Coll et al., 2017; de Waal and Preston, 2017); two of the core psychological processes of positive empathy are perspective taking and affect sharing – conceptual features of both positive empathy and empathy for pain (Morelli et al., 2015). To measure empathic perceptions, participants rated the *perceived positivity* of the uplifting experiences in the scenarios and the *perceived pleasure* in the scenario protagonists. To measure empathic affect, we focused on participants’ ability to affectively share in other people’s positive experiences. Specifically, participants rated *personal pleasure* for themselves and positive *other-directed empathic feelings* in response to scenario protagonists’ positive experiences. Finally, we explored a process model in which reductions in empathic perceptions statistically mediated the effects of acetaminophen consumption on reduced empathic affect to test whether the effects of acetaminophen on empathic affect are driven by respective impairments in empathic cognition, or not.

## Materials and Methods

### Participants

One-hundred and fourteen undergraduate students at Ohio State University (48 females; *M*_age_ = 18.8, *SD* = 1.31; 83 Whites, 12 Asian-Americans, 7 African-Americans, and 12 mixed race/others) participated for partial course credit toward their introductory psychology requirement. As reported before (Mischkowski et al., 2016), we determined sample size based on a power analysis of a previous study on the effects of acetaminophen on empathy for pain (Mischkowski et al., 2016, Study 1), which we had conducted in a similar population as the current study. The power analysis used a power criterion of (1 − *β*) = 0.80, which indicated that a mean cell size of *n* = 54 was sufficient to replicate significant effects of acetaminophen. We accounted for sample attrition by adding six participants to our sample, as four participants had dropped out in the previous study which had a sample size of about two thirds of the current study. Ultimately, seven participants dropped out during various stages of the current study. We retained these participants when they had provided sufficient data for a specific set of analyses. The Institutional Review Board at Ohio State University approved all experimental procedures.

### Materials and Procedures

After signing up, participants received an email informing them about contraindications for taking acetaminophen (e.g., currently taking acetaminophen or a drug containing acetaminophen, a history of liver disorder, having an allergic reaction to acetaminophen) and were asked to withdraw from the study when they met any of those contraindications. Furthermore, to facilitate drug absorption, participants refrained from consuming food at least 3 h before the start of the experiment.

Once in the laboratory, participants provided written informed consent and then were randomly assigned to consume 1,000 mg acetaminophen (*n* = 59) or a placebo (*n* = 55) in liquid form, using double-blind drug administration procedures. Solutions were stored in masked containers, preventing experimenters from learning about the nature of the solution, while participants were told they would be receiving either acetaminophen or placebo but not which type of solution. Following recommendations from previous research on absorption timing of acetaminophen (Møller et al., 2000; Randles et al., 2013; Durso et al., 2015), we waited 1 h before administering our measure of positive empathy and other dependent variables of interest. For additional procedural details and measures not germane to the current research questions, see Mischkowski et al. (2016).

#### Measures of Positive Empathy

To measure positive empathy, we modified an established empathy induction paradigm, which used written scenarios to measure empathy for pain (Bruneau et al., 2012). Specifically, we modified several scenarios to have positive instead of negative endings. Using written scenarios is an established means of inducing empathy; many empathy studies have relied on written descriptions to successfully induce empathy for both pleasurable and painful experiences (e.g., Perry et al., 2011; Bruneau et al., 2012; Andreychik and Migliaccio, 2015; Mischkowski et al., 2016). In random order, participants read and rated four scenarios depicting various protagonists having positive experiences:

1. John was on a hike with his girlfriend. He had an engagement ring in his pocket and at a beautiful overlook he proposed marriage. His girlfriend said that she would marry him and began crying. John held his new fiancée and kissed her.
2. Alex had wanted to ask Christy on a date for months. One day Alex walked up to her and asked her out. Christy said that she would be happy to go out with him. Afterwards, when he was alone, he pumped his fist.
3. Suzie had worked hard to get the job she has now. She can finally take care of her son and has almost saved enough to give him the gifts he wants for his birthday. Today in the office Suzie’s boss told her that she had earned a raise for the great job she was doing.
4. Lissa was excited to have a solo in her upcoming performance. Her father promised her that he would be there in the front row for the concert. When the concert comes, her father was there to watch. After the show, her dad told Lissa how proud he was to have such a talented daughter.

Separately for each of these scenarios, participants completed measures of positive empathic perceptions and affective empathy. Specifically, participants completed a one-item measure of *perceived positivity* (Berntson et al., 2011). On a scale from −5 (*extremely negative*) to 5 (*extremely positive*), participants rated the extent to which each scenario was positive or negative. Furthermore, participants completed a one-item measure of *perceived pleasure*, using a scale from −4 (*worst possible pain*) to 4 (*most possible pleasure*).^1^ In addition, participants completed a six-item measure of *personal pleasure*. On a scale from 1 (*not at all*) to 5 (*extremely*), participants rated the extent to which they felt *pleasure*, *delighted*, *uplifted*, *pleased*, *joyful*, and *cheerful* while imagining the feelings of each scenario protagonist. We averaged items separately for each scenario to create measures of personal pleasure (0.96 ≤ *α* ≤ 0.97). Finally, participants completed an established six-item measure of *other-directed empathic feelings* (Batson et al., 1995). On a scale from 1 (*not at all*) to 5 (*extremely*), participants indicated the extent to which they felt *sympathetic*, *warm*, *compassionate*, *soft-hearted*, *tender*, and *moved* while imagining the feelings of each scenario protagonist. We averaged items separately for each scenario to create measures of empathic feelings (0.92 ≤ *α* ≤ 0.93).^2^

#### Control Variables

To rule out baseline differences in affect between the acetaminophen and the placebo control conditions, we measured *baseline affect* and *arousal* using the respective one-item subscales of the Self-Assessment Manikin (SAM), an established and validated measure of state affectivity (Bradley and Lang, 1994; Lang et al., 1999). We administered the SAM items right after drug administration; because 1,000 mg liquid acetaminophen needs at least 45 min to exert pain relieving effects (Møller et al., 2000), it is unlikely that drug consumption influenced these measures. Thus, these measures served as measures of baseline affectivity. Participants indicated their feelings “right now,” using scales from 1 (affect: *unhappy*; arousal: *calm*) to 9 (affect: *happy*; arousal: *excited*).

Furthermore, we tested the possibility that acetaminophen changed positive empathy by changing *general affect*. Specifically, participants completed the Positive and Negative Affect Schedule (PANAS; Watson et al., 1988), indicating their feelings “right now.” Participants completed the PANAS at least 60 min after consuming acetaminophen or the placebo, before completing the empathy scenarios. We averaged the 10 positive and 10 negative affect items of the PANAS to create general measures of positive (*α* = 0.85) and negative affect (*α* = 0.82).

At the end of the study, participants also guessed whether they had consumed acetaminophen or the placebo to control for *perceived drug consumption*.

## Results

### Preliminary Analyses

To justify combining measures across scenarios, we tested whether scenario type moderated effects of acetaminophen relative to placebo on scenario measures. Specifically, we conducted repeated measures Analyses of Variances (ANOVAs) with drug condition (acetaminophen vs. placebo) as a between-subjects factor and scenario type as a within-subjects factor. Results justified combining measures across scenarios, as scenario type did not moderate effects of acetaminophen relative to placebo on perceived positivity, perceived pleasure, personal pleasure, or empathic feelings (*F*s ≤ 1.01, *p*s ≥ 0.388, $\eta_{p}^{2}$ ≤ 0.013).

Furthermore, we confirmed that the scenarios induced an experience of positive empathy in our participants. For this manipulation check, we limited our analyses to participants in the placebo group and used one-sample *t*-tests to confirm that participants reported elevated empathic perceptions and affect relative to neutral response scale points when reading scenarios. As expected, participants perceived all scenarios as quite positive (3.09 ≤ *M*s ≤ 4.42, 1.17 ≤ *SD*s ≤ 1.43) and perceived scenario protagonists to experience moderate to a lot of pleasure (2.63 ≤ *M*s ≤ 3.35, 0.90 ≤ *SD*s ≤ 1.55); perceived positivity and pleasure were significantly elevated relative to the neutral scale points (i.e., 0) (*t*s ≥ 10.83, *p*s < 0.001, *d*s ≥ 1.69). Similarly, participants experienced somewhat to a lot of personal pleasure (3.12 ≤ *M*s ≤ 3.66, 1.07 ≤ *SD*s ≤ 1.16) and a little to somewhat other-directed empathic feelings (2.50 ≤ *M*s ≤ 3.12, 0.97 ≤ *SD*s ≤ 1.12) when reading the scenarios; personal pleasure and empathic feelings were significantly elevated relative to the scale point indicating the absence of any empathic affect (i.e., 1) (*t*s ≥ 8.69, *p*s < .001, *d*s ≥ 1.47). We thus conclude that our scenarios were able to induce empathic perceptions and affect when reading about the positive experiences of other people.

Additionally, we checked whether participants were blind to drug condition. Despite double-blind drug administration procedures, 31.86% of participants correctly indicated that they had consumed acetaminophen, and 30.09% of participants correctly indicated they had consumed the placebo, which, in sum, was significantly above chance (50%) as reported previously (Mischkowski et al., 2016, Study 2). However, the participants who correctly identified their drug status did not show any differences in empathic perceptions or affect relative to the participants who incorrectly identified their drug status (*F*s ≤ 0.43, *p*s ≥ 0.513, $\eta_{p}^{2}$ ≤ 0.004), nor did the (mis-)identification of drug status moderate the effect of actual drug condition on any measures of empathic perceptions or affect (*F*s ≤ 1.10, *p*s ≥ 0.297, $\eta_{p}^{2}$ ≤ 0.010). We nevertheless controlled for perceived drug consumption by including perceived drug consumption as a statistical covariate in all subsequent analyses. Unless indicated otherwise, however, controlling for perceived drug consumption did not substantially change results.

Finally, we ruled out that participants differed in baseline affect and arousal depending on whether they had been assigned to the acetaminophen or the placebo condition. As would be expected with successful randomization, participants in the acetaminophen condition reported levels of baseline general affect (*M* = 6.57, *SE* = 0.17) similar to participants in the placebo condition (*M* = 6.58, *SE* = 0.16). Participants in the acetaminophen condition also reported levels of baseline arousal (*M* = 3.92, *SE* = 0.24) similar to participants in the placebo condition (*M* = 3.85, *SE* = 0.23). Participants in the acetaminophen and placebo conditions did not significantly differ in baseline affect and arousal (*F*s ≤ 0.05, *p*s ≥ 0.831, $\eta_{p}^{2}$ ≤ 0.0004).

### Effects of Acetaminophen on Positive Empathy

**Figure 1:**
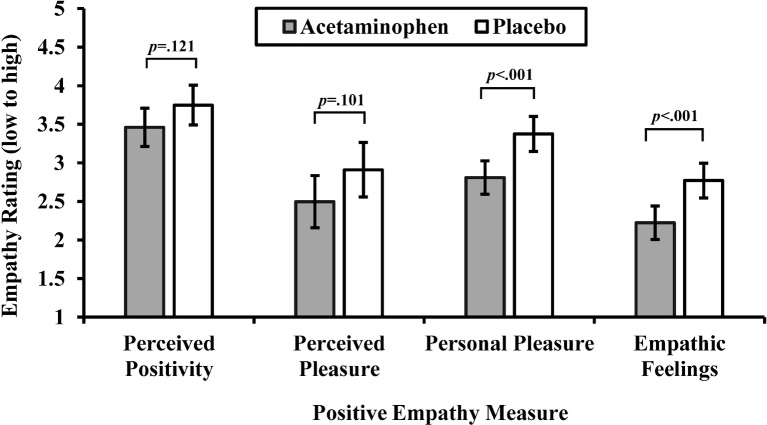
Mean ratings on positive empathy measures by drug condition (acetaminophen vs. placebo). Perceived positivity is rated on a scale from 5 (*extremely negative*) to 5 (*extremely positive*); perceived pleasure is rated on a scale from −4 (*worst possible pain*) to 4 (*most possible pleasure*); personal pleasure and empathic feelings items are rated on scales from 1 (*not at all*) to 5 (*extremely*). Capped vertical bars denote 95% confidence intervals (CIs). Means and CIs are adjusted for perceived drug consumption.

We used ANOVAs with drug condition (acetaminophen vs. placebo) as a between-subjects factor to test whether acetaminophen reduced perceived positivity, perceived pleasure, personal pleasure, and empathic feelings. See Figure 1 for a graphical depiction of effects of drug consumption on positive empathy measures. As predicted, acetaminophen relative to placebo reduced personal pleasure, *F*(1,110) = 12.38, *p* < 0.001, $\eta_{p}^{2}$ = 0.101, and empathic feelings, *F*(1,110) = 11.67, *p* < 0.001, $\eta_{p}^{2}$ = 0.096. In contrast, acetaminophen relative to placebo did not significantly reduce perceived positivity, *F*(1,110) = 2.44, *p* = 0.121, $\eta_{p}^{2}$ = 0.022, or perceived pleasure, *F*(1,110) = 2.74, *p* = 0.101, $\eta_{p}^{2}$ = 0.024. According to these results, acetaminophen reduced the empathic emotional response when reading about other people having positive experiences but did not affect perceptions about these people’s positive experiences.

### Empathic Perceptions Needed as Mediators?

Though acetaminophen did not significantly affect perceptions of target’s positivity or pleasure, we tested a mediation model in which empathic perceptions mediated the effect of acetaminophen on empathic affect, as the indirect effect *via* a mediator can still be significant despite an independent variable not exerting a significant effect on the mediator variable (Hayes, 2018). Specifically, we tested in two sets of mediation analyses whether reduced perceptions of positivity and pleasure accounted for the effects of acetaminophen on reduced empathic affect, specifically personal pleasure and other-directed empathic feelings. To test for indirect effects, we drew 5,000 bootstrapping samples to construct bias-corrected 95% confidence intervals around these effects, using the PROCESS macro in SPSS (Hayes, 2012). Because perceived positivity correlated with perceived pleasure, *r*(112) = 0.57, *p* < 0.001, we entered both variables as parallel mediators into both mediation models. This approach allowed us to test for the combined indirect effect through perceived positivity and pleasure, as well as for the independent indirect effects through these variables. However, neither perceived positivity [−0.004, 0.112], nor perceived pleasure [−0.038, 0.046], nor both measures in combination [−0.009, 0.112] mediated the effect of acetaminophen on reduced personal pleasure. Similarly, neither perceived positivity [−0.018, 0.067], nor perceived pleasure [−0.033, 0.038], nor both measures in combination [−0.015, 0.070] mediated the effect of acetaminophen on other-directed empathic feelings. These findings suggest that empathic perceptions do not account for the effect of acetaminophen on decreased empathic affect.

### Accounting for General Affect

Finally, we tested whether general positive or negative affect accounted for the effect of acetaminophen relative to placebo on positive empathy measures. Consuming acetaminophen relative to placebo did not change general positive or negative affect measured 1 h after drug administration (just prior to reading the empathy scenarios). Participants in the acetaminophen condition did not differ in positive affect (*M* = 2.53, *SE* = 0.10) from participants in the placebo group (*M* = 2.62, *SE* = 0.11), *F*(1,110) = 0.37, *p* = 0.545, $\eta_{p}^{2}$ = 0.003. Similarly, participants in the acetaminophen condition did not differ in negative affect (*M* = 1.27, *SE* = 0.05) from participants in the placebo group (*M* = 1.27, *SE* = 0.05), *F*(1,110) = 0.01, *p* = 0.933, $\eta_{p}^{2}$ = 0.000. Again, we used non-parametric bootstrapping to test whether positive or negative affect statistically accounted for the effect of acetaminophen consumption on reduced positive empathy measures. Positive affect did not statistically account for any of the effects of acetaminophen relative to placebo on perceived positivity [−0.042, 0.045], perceived pleasure [−0.046, 0.088], personal pleasure [−0.066, 0.140], or empathic feelings [−0.063, 0.130]. Similarly, negative affect did not statistically account for any of the effects of acetaminophen relative to placebo on perceived positivity [−0.115, 0.068], perceived pleasure [−0.146, 0.087], personal pleasure [−0.027, 0.032], or empathic feelings [−0.028, 0.032]. In summary, effects of acetaminophen on positive empathy measures seem to be independent of general affect.

## Discussion

As predicted, acetaminophen reduced positive empathy. When reading scenarios about various protagonists having pleasurable experiences, participants under the influence of acetaminophen experienced less empathic affect compared to participants who had consumed a psychologically inert placebo. In contrast, acetaminophen did not reduce empathic perceptions, and an exploratory mediation model in which empathic perceptions accounted for the effect of acetaminophen on empathic affect failed to receive empirical support. Acetaminophen’s effects on positive empathy measures held regardless of whether participants assumed that they had consumed acetaminophen or a placebo. Finally, general positive or negative affect did not account for acetaminophen’s effects on positive empathy.

These findings have important theoretical and practical implications and point toward promising future research directions. First and foremost, these results add to our understanding of the mechanism underlying the experience of positive empathy. Our research supports the idea that experiences of positive and negative empathy at least partly rely on the same neurochemical and psychological processes, as acetaminophen also reduces empathy for pain (Mischkowski et al., 2016). Though different forms of empathy likely share common psychological processes, such as mentalizing about other people’s feelings, affect sharing, or prosocial motivation (Gallese and Goldman, 1998; Decety and Jackson, 2004; Singer, 2009; Zaki and Ochsner, 2012; Morelli et al., 2015; de Waal and Preston, 2017; Lamm et al., 2017), the extent to which positive empathy shares common and distinct features with negative empathy, as well as with personal pleasure or pain, remains an emerging subject of investigation (Morelli et al., 2015).

The finding that acetaminophen impairs affective but not cognitive empathy suggests that acetaminophen impairs a neurochemical mechanism that mediates the affective susceptibility to other people’s emotional experiences rather than understanding the emotional impact of these experiences for others. These effects are consistent with other findings from our lab, indicating that acetaminophen reduces pain and compassion in response to other people’s pains more than the perceptions of pain intensity (Mischkowski et al., 2016). Furthermore, acetaminophen reduces affective reactivity to other’s negative or positive experiences, while keeping cognitive evaluations of these experiences largely intact (Durso et al., 2015; Roberts et al., 2017). This finding supports the idea that empathy relies on the affective sharing in other people’s positive or negative experiences, an idea that is consistent with simulation theories of empathy (de Waal and Preston, 2017).

Furthermore, our findings suggest that affective mechanisms recruited by physical pain also play a role in positive empathy. Acetaminophen reduces physical pain, and previous research has directly or indirectly suggested a role of physical pain in the experience of empathy for pain (for reviews, see Gallese and Goldman, 1998; Decety and Jackson, 2004; Singer, 2009; de Waal and Preston, 2017; but see Decety, 2010; Zaki et al., 2016; Lamm et al., 2017); in contrast, potential neurochemical overlap between physical pain and positive empathy has not received much attention. Thus, our research suggests there may be a deeper neuronal connection between physical pain and positive empathy than previously assumed. Areas of the AI and the dACC are candidates for representing such a shared basic mechanism, as these areas seem to be part of a larger neuronal network representing basic aspects of affect awareness and regulation (Craig, 2009; Etkin et al., 2015). However, more research on the exact neuronal and psychological substrate of the effect of acetaminophen on positive empathy is needed.

Speculations on the neurochemical substrates of our findings are also limited by what we know about acetaminophen’s exact neurochemical mechanism of action which remains contentious. Past research has suggested a prostanoid mechanism (Flower and Vane, 1972), while more recent experimental evidence suggests that a combination of endocannabinoid, vanilloid, and serotonergic neurochemical mediators may also mediate effects of acetaminophen (Mallet et al., 2017). A central serotonergic mechanism would be a promising candidate to explain effects of acetaminophen on both positive and negative empathy (Mischkowski et al., 2016), as dispositional variations in serotonergic neurotransmission affect sensitivity to both positive and negative emotions of other people (Way and Keaveney, 2018). Such a finding is consistent with neurodevelopmental theories suggesting that the same physiological or genetic factors shape increased susceptibility to both positive and negative experiences (Boyce and Ellis, 2005; Belsky and Pluess, 2009). Though acetaminophen or its psychoactive metabolites do not directly interact with serotoninergic receptors, depleting serotoninergic activity in the brain reduces the analgesic effects of acetaminophen (Mallet et al., 2017). Psychological effects of acetaminophen, including on positive empathy, may rely on a similar serotoninergic mechanism, though such a mechanism awaits to be demonstrated conclusively.

Because of their novelty, the present findings need replication and extension. The current study employed a measure of positive empathy using hypothetical scenarios. Hypothetical situations have been used in the past to successfully induce both positive and negative empathy (Perry et al., 2011; Bruneau et al., 2012; Mischkowski et al., 2016). However, to increase ecological validity of our findings, it would be desirable to replicate findings, using real situations to induce positive empathy, e.g., when watching somebody else winning money or receiving pleasant touch (e.g., Mobbs et al., 2009; Ebisch et al., 2011; Braams et al., 2013; Apps and Ramnani, 2014; Lamm et al., 2015). To evaluate the extent to which the scenarios in this study induced positive empathy, it would also have been desirable to include neutral (control) scenarios. Based on our analyses, we are confident that our scenarios induced the experience of positive empathy and that acetaminophen reduced positive empathy. However, without control scenarios, it is difficult gauge the relative reduction in positive empathy as a result of acetaminophen consumption. Furthermore, to differentiate effects of acetaminophen on vicarious and first-hand experience of pleasure, it would be desirable to use functional imaging, preferably in combination with multivoxel pattern analysis to identify shared and distinct networks of neuronal activation (Corradi-Dell’Acqua et al., 2011; Krishnan et al., 2016). Such an analytic approach would also provide more leverage to identify the features of positive empathy that are shared with or distinct from other types of affective experiences.

Finally, our findings have important practical implications. Positive empathy provides part of the “social glue” from which interpersonal bonds are built and strengthened (Morelli et al., 2015). As such, taking pleasure from the good fortune of others fosters interpersonal connection, trust, and – ultimately – prosocial behavior (Reis et al., 2010; Morelli et al., 2014; Andreychik and Migliaccio, 2015), thus providing important societal benefits. These benefits have to be viewed in the context of the amount of people regularly consuming acetaminophen. An estimated quarter of all US American adults take a drug containing acetaminophen every week (Kaufman et al., 2002). It is thus possible that the pervasive use of acetaminophen among Americans may substantially reduce these benefits. However, there is currently no research on the relationship between acetaminophen usage and reduced prosocial behavior in the USA. This research gap needs filling.

Overall, the present research shows that acetaminophen reduces empathy for the pleasurable experiences of other people. These findings not only constitute an important step forward in our understanding of the affective mechanisms underlying the experience of positive empathy but also raise concern about the societal impact of excessive acetaminophen consumption.

## Ethics Statement

The Ohio State Office of Responsible Research Practices (ORRP) approved all experimental procedures. All participants provided informed written consent.

## Author Contributions

DM, JC, and BW developed hypotheses and designed the study. DM conducted data analysis. DM, JC, and BW wrote the manuscript.

### Conflict of Interest Statement

The authors declare that the research was conducted in the absence of any commercial or financial relationships that could be construed as a potential conflict of interest.

## Footnotes

^1^We worded this item in a bipolar manner, ranging from *pain* to *pleasure*, because we also used this item in the same study to measure empathic perceptions of pain (Mischkowski et al., 2016). To make answering empathy questions easier for our participants, we decided to cover empathic perceptions of both pleasure and pain in one overarching item.

^2^Due to a programming error, 35 participants did not complete the second empathy scenario. Therefore, aggregated positive empathy measures only include scenarios 1, 3, and 4 for these participants.
